# Associations between exposure to nutrition, WASH interventions and children’s academic performance in Ethiopia: a systematic review and meta-analysis

**DOI:** 10.1186/s12889-025-26107-4

**Published:** 2026-01-12

**Authors:** Yimer Mihretie Adugna, Abebe Ayelign, Tadesse Alemu Zerfu

**Affiliations:** 1 Center for Food Science and Nutrition, Addis Ababa University, P.O. Box 1176, Addis Ababa, Ethiopia; 2 International Food Policy Research Institute (IFPRI), P.O. Box 5689, Addis Ababa, Ethiopia

**Keywords:** Academic performance, Ethiopia, Nutrition interventions, Systematic review, Water, sanitation, and hygiene interventions, WASH

## Abstract

**Background:**

Poor nutrition and inadequate WASH (water, sanitation, and hygiene) practices significantly impact children’s health, nutrition, and cognitive development, especially in low-income settings. These factors further aggravate the incidence of undernutrition, weaken the immune system, increase susceptibility to illnesses and reduce cognitive performance. Evidence on the effectiveness of existing WASH interventions is needed.

**Objective:**

This review evaluated the effectiveness of nutritional and WASH interventions on the academic performance of children in Ethiopia.

**Methods:**

A systematic search of Cochrane, DOAJ, Google Scholar, and PubMed (2010–2024) was conducted using MeSH terms and keywords related to WASH. Two independent reviewers screened studies and extracted data. Eligible studies included cross-sectional and cohort studies on Ethiopian schoolchildren with quantifiable academic outcomes. The JBI SUMARI was used to assess bias, and the GRADE approach was used to evaluate evidence quality. The meta-analysis used a random-effects model in Stata and reported pooled RRs with 95% CIs. Subgroup and sensitivity analyses examined moderators such as study design, intervention type, and sample size.

**Results:**

A total of 19 studies, 16 cross-sectional (*n* = 16) and three prospective (*n* = 3) cohort studies with a total of 9034 participants, were included. The random effects model revealed a significant improvement in academic performance among students receiving both nutrition and WASH, with a pooled large positive effect size of 2.05 (95% CI: 1.26, 2.28; I2=). In the subgroup meta-analysis, the effect of the intervention was more positive among those who skipped breakfast (3.47, 95% CI: 0.47, 6.47), chronic iodine deficiency (4.49, 95% CI: 4.08, 4.90), food insecurity (2.810, 95% CI: 1.281, 4.339), and underweight (0.61, 95% CI: 0.46, 0.75).

**Conclusion:**

Despite moderate variability and some risk of bias, the evidence supports the integration of comprehensive nutrition and WASH programs into school health initiatives. Future research should focus on long-term effects and cost-effectiveness.

**Trial registration:**

This systematic review and meta-analysis were registered in the International Prospective Register of Systematic Reviews (PROSPERO) under the ID CRD42024567265.

**Supplementary Information:**

The online version contains supplementary material available at 10.1186/s12889-025-26107-4.

## Key message

**What is already known?**

• Malnutrition (including undernutrition), breakfast skipping, iodine deficiency, poor hand washing practices, inadequate sanitation, and related diseases are key factors that impact cognitive development and academic performance.

• Previous studies in low-income countries, including Ethiopia, have confirmed these associations.

**What are the findings of this study?**

• Few or no high-quality studies have been conducted to evaluate the effects of nutrition and wash interventions on Ethiopian schoolchildren's academic performance.

• While many studies have examined nutrition or WASH separately, none have explored their combined effects—particularly among school-age children in Ethiopia.

**What are the implications of the new findings?**

• Our research revealed that WASH and nutrition have a major influence on Ethiopian students' academic achievement.

• Skipping breakfast and having a chronic iodine shortage have detrimental effects on learning, impairing focus and cognitive function.

**Supplementary Information:**

The online version contains supplementary material available at 10.1186/s12889-025-26107-4.

## Introduction

Malnutrition and poor water, sanitation, and hygiene (WASH) conditions are significant public health challenges in many low-income countries, including Ethiopia [1]. These issues profoundly impact the health and development of particularly vulnerable children. Malnutrition has been shown to impair physical and cognitive development, leading to stunted growth, weakened immunity, and reduced learning capacity. Inadequate WASH facilities further heighten the risk of infectious diseases, exacerbating malnutrition and its consequences [2]. These health challenges directly affect children’s academic performance by contributing to frequent school absences, decreased concentration, and decreased cognitive ability [3].

Malnutrition and poor sanitation are significant challenges that impact children’s health and their ability to succeed in school. Research consistently shows that nutrition interventions, such as school-based feeding programs and micronutrient supplementation, can improve children’s health and cognitive function, leading to better academic performance [4–6]. Likewise, WASH (Water, Sanitation, and Hygiene) interventions, including access to clean water, improved sanitation facilities, and hygiene education, are essential for reducing waterborne diseases and enhancing overall health [7].

Despite the well-documented benefits of these interventions on children’s health, their direct impact on academic performance remains underexplored, especially in Ethiopia. Given the widespread malnutrition and inadequate sanitation in the country, understanding how these factors affect children’s ability to perform well in school is crucial.

Considering the complex relationships among nutrition, health, and education, a systematic review of the available evidence on the effectiveness of these interventions on academic performance is essential [8, 9]. This review aims to address this gap by synthesizing findings from various studies to provide a comprehensive understanding of how nutrition and WASH interventions influence educational outcomes in Ethiopian school-age children. Therefore, this systematic review and meta-analysis was conducted to assess the effects of nutrition and WASH interventions on academic performance among school-age children in Ethiopia, offering valuable insights to inform future policies and interventions aimed at improving both health and educational outcomes.

## Materials and methods

### Data sources and search strategy

A systematic search for relevant literature was conducted in European PubMed, the Cochran clinical trial, DOAJ, Google Scholar, and PubMed via key words and medical subject headings (MeSHs) (Table 1). Many studies found in SCOPUS and Web of Science were duplicates of those retrieved from Cochrane, PubMed, DOAJ and Google scholar. Given our focus and the substantial overlap, we determined that Cochrane, PubMed and DOAJ were sufficient for this review. The search was confined to the period from January 2010 to April 2024. Moreover, gray literature and common institutional repository (AAU, HU, JU) libraries were extensively searched for unpublished works. The search was performed by the following authors: YM, AA, and TAZ, using the key words, or MeSH terms, using Boolean operators.

**Table 1:** Search strategy and results from databases on nutrition, WASH interventions, and academic performance in school-aged children in Ethiopia

Database	Search Strategy	Total Results	Eligible Studies
DOAJ	effectiveness OR Impact OR “Associated factors AND Nutrition AND (WASH OR wash-in) AND Intervention AND (School-aged OR Students OR School students OR Children) AND academic AND (performance OR Success) AND Ethiopia	1000	6
PubMed	(((Effectiveness [Title/Abstract] AND Nutrition [Title/Abstract]) AND ((WASH [Title/Abstract] OR “wash-in” [Title/Abstract]) OR WASH [MeSH Terms])) AND Intervention [Title/Abstract]) AND ((School-aged [Title/Abstract] OR Students [Title/Abstract]) OR (“School students” [Title/Abstract] OR Children [Title/Abstract])) AND (academic [Title/Abstract] AND (performance [Title/Abstract] OR Success [Title/Abstract])) AND (Ethiopia [Title/Abstract] OR “Ethiopia” [MeSH Terms])	7217	10
Cochrane Library	((Effectiveness AND Nutrition) AND ((“Water Sanitation Hygiene”) OR WASH) AND Intervention) AND ((School-aged OR Students) OR (“School students” OR Children)) AND (academic AND (performance OR Success)) AND Ethiopia	9	3

The study was organized and reported on the basis of the Preferred Reporting Items for Systematic Reviews and Meta-Analyses (PRISMA) guidelines [10] (Supplemental Tables 2 & 3). The protocol for this systematic review and meta-analysis was registered in the International Prospective Register of Systematic Reviews (PROSPERO; ID: CRD42024567265). Minor revisions—such as clarifying the wording of the inclusion criteria and updating the search strategy to incorporate recent literature—were implemented to enhance transparency and comprehensiveness, without altering the overarching objectives or methodological framework of the review.

### Eligibility criteria

All primary studies that included school students aged 5–18 years attending primary or secondary educational institutions in Ethiopia, published from 01/01/2010 to 30/01/2025 were included. The review also included studies that involved both male and female students from various socioeconomic backgrounds. Additionally, studies that involved nutrition interventions, such as school feeding programs, micronutrient supplementation, dietary education, and other programs designed to increase the nutritional status of children, as well as WASH interventions, including the provision of clean water, construction or renovation of sanitation facilities, and hygiene education programs, were included if they evaluated the impact of these interventions on academic performance. Secondary reports and studies that focused exclusively on special populations (e.g., children with severe disabilities) or adult participants were excluded to maintain a focus on the typical school-age population.

### Patients and public involvement

This study did not directly involve patients or the public in its design, analysis, or interpretation. However, insights from educators, public health officials, and community members were considered when formulating research questions and discussing implications.

### Data collection

All available published and unpublished articles were retrieved from online databases via nested knowledge software. Two reviewers (YM and AA) independently extracted data from each report. Any discrepancies between the reviewers were resolved through discussion. The data collection process was supported by the automation features of the Nested Knowledge platform, which facilitated article screening and data management.

### Screening and data extraction

After all the available published and unpublished studies were collected, duplicates were removed on the basis of nested knowledge [11]. The titles and abstracts of 8226 studies were subsequently reviewed. The full-text review was performed for relevant articles remaining after the title and abstract were reviewed. Finally, articles with no variable interest and low quality were excluded. After the title, abstract, and full-text were reviewed, all relevant studies were extracted by using the prepared Microsoft Excel data extraction format. The data extraction form included the first author’s name, year of publication, study design, effect size, confidence interval, standard error, and sample size. The format will also include the exposure (predictor variables) and outcome variable academic performance. Two reviewers, AA and TAZ, extracted the data, and any disagreement on the extracted data was resolved by rechecking the article to reach a common conscience. Inconsistent and incomplete data were removed after data extraction.

### Variables

#### Outcome of interest

The primary outcome of this review is the academic performance of students, which can be measured and captured in various ways. These include standardized test scores, grades or GPA, school attendance rates, cognitive assessments, and school completion rates.

#### Predictor variables

Predictor variable study setting, sample size, region, community or facility-based, dietary diversity, food insecurity, school feeding programs, and meal skipping, which are reported in studies, were included.

#### Effect measurement

In this systematic review and meta-analysis, the outcomes were reported via standardized effect sizes since the included studies used a variety of measurement types, such as odds ratios, beta coefficients, regression coefficients, and mean differences. Standardization allowed for meaningful comparison and synthesis across the different outcome measures.

#### Quality assessment

The quality of all the studies was assessed through critical appraisal via the Joanna Briggs Institute Meta-Analysis of Statistics Assessment and Review Instrument (JBI-SUMARI) [12], which is already integrated into nested knowledge. This critical appraisal tool evaluates aspects such as sample representativeness, appropriate recruitment of participants, sufficient description of participants, use of standard measurement tools and their reliability, appropriate statistical analysis with sufficient coverage of the identified sample size, and identification of subgroup or confounding variables. On the basis of this critical appraisal checklist, studies scoring greater than or equal to 7 out of 10 were considered high quality, whereas those scoring less than 7 were considered low quality. Consequently, all relevant studies had scores above 7. Additionally, the certainty of the evidence was assessed via the GRADE standard EtD templates from GRADE pro, which provided high certainty of evidence for the data [13].

### Data analysis

After data extraction in Microsoft Excel, the dataset was transferred to STATA version 16 for meta-analysis. The key characteristics of the included studies, such as the first author’s name, publication year, study location, study design, and sample size, are summarized in a table. The effect sizes (e.g., odds ratios, mean differences, or regression coefficients), along with standard errors and standard deviations from each primary study, were used to calculate the pooled effect size for academic performance. The pooled effect size was visually represented via a forest plot based on Cohen’s d: lower effect < 0.2, medium effect = 0.5, and high effect ≥ 0.8 [14].

Heterogeneity among the studies’ risk ratios was assessed via the I² statistic, with a *p* value of less than 0.05 indicating significant heterogeneity. A random-effects meta-analysis model was employed to account for heterogeneity at the 5% significance level. To explore potential sources of heterogeneity, a meta-regression analysis was conducted.

Publication bias was evaluated via Egger’s and Begg’s correlation tests at the 5% significance level, as well as via funnel plots. Additionally, the trim-and-fill method was applied to quantify the effect size of missed studies and enhance the robustness of the meta-regression findings. To address random variations between individual study estimates, subgroup analyses were performed on the basis of study design and study settings. Finally, the associations between predictor variables and outcome variables were expressed as relative risk ratios (RRs) with 95% confidence intervals.

### Ethics approval statement

Ethical clearance was obtained from the College of Natural and Computational Sciences Institutional Review Board (CNS-IRB) at Addis Ababa University (Approval Code: CNCSDO/515/15/2023). Consent to participate was not applicable, as this study is a systematic review and meta-analysis using previously published data.

## Results

### Study selection

**Fig. 1:**
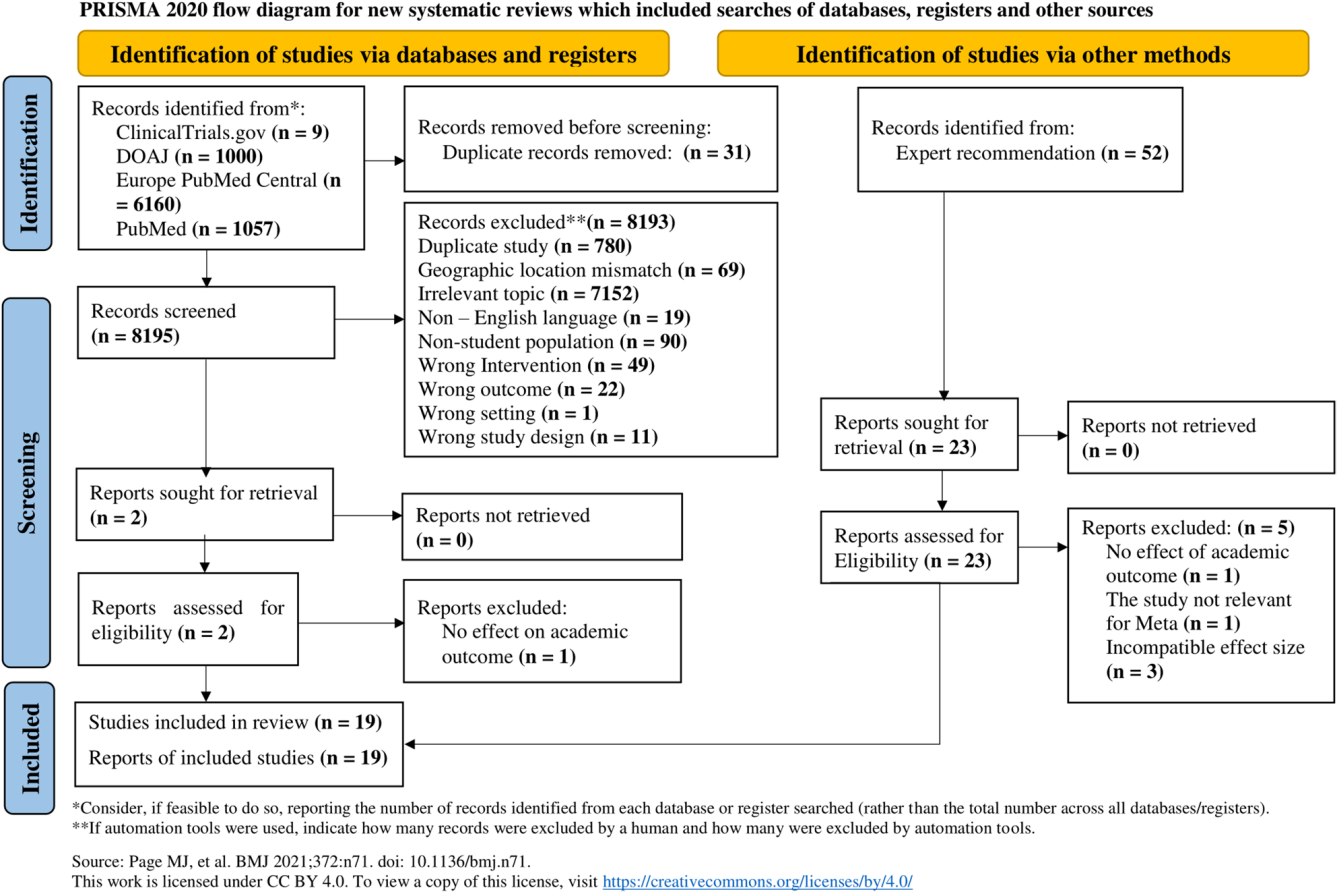
PRISMA flow chart showing the selection of included studies

A total of 8,226 studies were identified through an electronic search in European PubMed, Cochrane Clinical Trials, DOAJ, Google Scholar, PubMed, and gray literature, covering publications from January 2010 to April 2024. After the initial screening, 31 duplicate studies were removed. During the subsequent screening process, 8,195 studies were excluded. Additionally, 28 studies identified through other searches (e.g., expert recommendations) were excluded. At the eligibility assessment stage, 5 studies were excluded. Ultimately, 16 cross-sectional studies and 3 prospective cohort studies met the eligibility criteria and were included in this systematic review and meta-analysis. While we made strong efforts to identify RCTs and longitudinal studies, most available studies in Ethiopia focused on different populations, such as under-five children. As a result, we relied on cross-sectional studies to explore the associations between nutrition, WASH, and academic performance among school-age children (Fig. 1).

### Inclusion and exclusion criteria

Studies were included if they (1) involved school-aged children or adolescents (2), assessed nutritional status or related academic outcomes (3), were peer-reviewed articles published in English, and (4) employed quantitative or mixed-methods designs. Excluded studies were those focusing solely on adults, reviews, editorials, conference abstracts, or studies lacking relevant outcome data.

### Characteristics of the reviewed studies

Among the 19 included studies, 16 had a cross-sectional design, and 3 were prospective cohort studies. The studies were performed in different regions and zones of Ethiopia: two in Addis Ababa, five in the Amhara region, five in Oromia, one in the Sidama region, five in SNNPR, one in the SWEPR, and one in Somaliland. All the studies were published between 2010 and 2024, with sample sizes ranging from 131 [15] to 1205 participants [16]. Finally, 19 studies were considered for estimating the pooled odds ratio of academic performance, with a total sample size of 9034 (Table 2).

**Table 2:** Characteristics of studies included in the systematic review and meta-analysis of pooled effect sizes on the academic performance of students in Ethiopia

Study	Study region	Study settings	Study design	Effect size (mean)	Sample size
Abebe, Frehiwot (2017) [17]	Oromia Region	primary schools	Crossectional	0.48	630
Abebe, Lulu (2022) [18]	SNNPR	primary schools	Crossectional	2.76	848
Admasie, Amha (2013) [19]	Amhara Region	primary schools	Crossectional	0.58	601
Asfaw, Agize (2020) [20]	SWEPR	primary schools	Prospective Cohort Study	4.49	652
Asmare, Biachew (2018) [21]	Amhara Region	primary schools	Crossectional	0.63	442
Bekri Mohammed (2023) [22]	Addis Ababa	primary schools	Prospective Cohort Study	2.37	644
Degarege, Abraham (2022) [16]	Amhara Region	primary schools	Crossectional	0.92	1205
Desalegn, Tsion A (2021) [23]	Sidama region	primary schools	Prospective Cohort Study	2.4	480
Eniyew, Tigist (2019) [24]	Amhara Region	primary schools	Crossectional	7.61	399
Feye, Dereje (2023) [25]	Oromia Region	high schools	Crossectional	2.22	422
Haile, Demewoz (2016) [15]	Oromia Region	primary schools	Crossectional	0.53	131
Honja Kabero, Tesfaye (2021) [8]	SNNPR	primary schools	Crossectional	−0.27	178
Muluken Ayehu, Solomon (2021) [26]	Addis Ababa	primary schools	Crossectional	1.796	324
Senapathy, Marisennayya (2023) [27]	SNNPR	primary schools	Crossectional	2.51	306
Seyoum, Dejene (2019) [9]	Oromia Region	Primary schools	Crossectional	0.57	362
Tamiru, Dessalegn (2016) [28]	Oromia Region	Primary schools	Crossectional	2.81	434
Terefe, Birhanu (2021) [29]	Amhara Region	primary schools	Crossectional	6.51	308
Wolde, Tsedeke (2019) [30]	SNNPR	primary schools	Crossectional	0.025	378
Zenebe, Mastewal (2018) [31]	SNNPR	primary schools	Crossectional	−0.79	290

### Risk of bias in studies

To assess the internal validity of the studies, after title and abstract screening, full-text screening was conducted for the included studies via the JBI SUMARI Critical Appraisal Checklist [17]. This checklist contains 8 questions for cross-sectional studies and 11 questions for RCT (cohort) studies, with responses rated as “0” (high risk – may be excluded) or “1” (low risk – may be included). For inclusion, cross-sectional studies needed to score at least 6 points out of 8 (75%), and RCT (cohort) studies needed to score at least 6 points out of 11 (approximately 55%) [18].

In this meta-analysis, of the 15 cross-sectional studies, 7 scored 8/8, 6 scored 7/8, and 2 scored 6/8; all were included. Similarly, for the RCT (cohort) studies, all four studies scored 11/11 and were included. Additionally, the certainty of the evidence was assessed via the GRADE standard EtD templates from GRADEpro [13], which provided high certainty of evidence for the data.

### Outcome operationalization

Due to the variability in outcome measures across studies, primary outcomes were categorized based on conceptual similarity. Academic performance indicators (e.g., test score, grades) were grouped under the broader theme of “academic performance.” Similarly, nutritional status was operationalized using anthropometric indicators such as stunting, underweight, as well as dietary patterns and other classifications of nutritional condition. These outcomes were reported and analyzed using standardized definitions provided in the original studies. Effect sizes, including odds ratios, mean differences, and beta coefficients, were converted to a common standardized effect size for synthesis and comparison.

### Academic scores among students in Ethiopia

The pooled academic performance score among students in Ethiopia, synthesized from 19 studies, was 2.05 (95% CI: 1.26, 2.28). A random-effects model was applied due to the presence of significant heterogeneity between studies (I² = 97.0%, *p* < 0.001). The variability observed reflects substantial differences across studies in terms of methodology, population characteristics, and contextual factors.

**Fig. 2:**
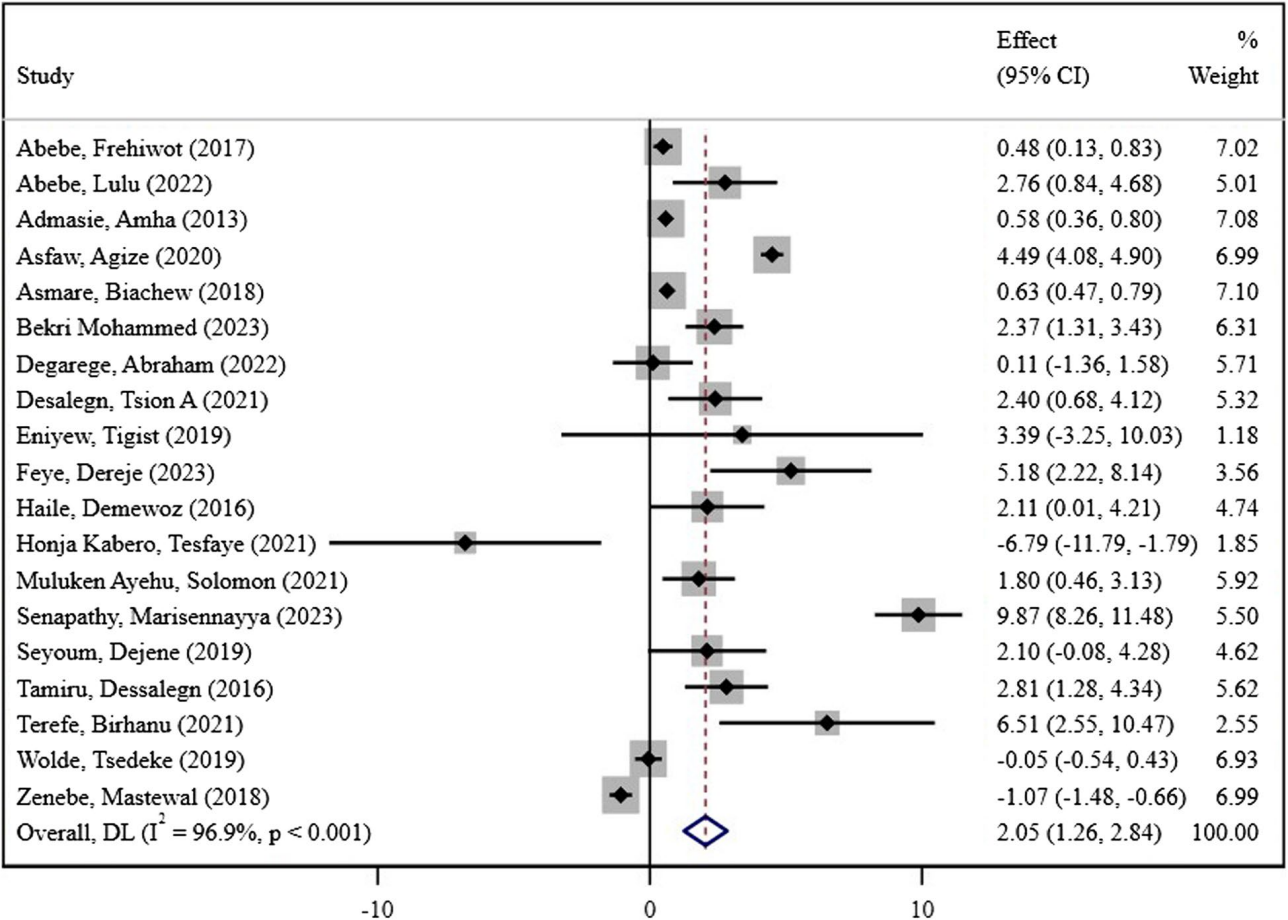
Forest plot of the effect size of the academic performance of students in Ethiopia

The lowest effect size was − 6.79, reported by Honja Kabero and Tesfaye (2021) [8], and the highest was 9.87, reported by Senapathy and Marisennayya (2023) [27]. This range highlights the diverse influences on Ethiopian students’ academic outcomes (Fig. 2).

### Subgroup analysis by geographical region

Due to the significant heterogeneity observed across studies, a subgroup analysis was conducted based on geographic region. The analysis revealed notable differences in pooled prevalence estimates: 2.25 (95% CI: 0.657, 3.850) in the Oromia Region [9, 15, 20–22], 2.15 (95% CI: 1.319, 2.977) in Addis Ababa [23, 24], 0.631 (95% CI: 0.298, 0.963) in the Amhara Region [16, 25–28], and 1.54 (95% CI: −1.11, 4.19) in the SNNPR [8, 19, 29–31], whereas the individual effect sizes were 4.49 (95% CI: 4.08, 4.90) in the SWEPR [32] and 2.05 (95% CI: 1.256, 2.835) in the Sidama Region [33], as shown in Table 3.

The higher pooled prevalence observed in some regions may reflect deeper structural and contextual differences. In areas with elevated prevalence, higher poverty rates and limited access to nutritious food may lead to stronger associations between malnutrition and poor academic outcomes. Furthermore, cultural practices related to child feeding, parental involvement in education, and gender roles may also influence both nutritional status and school performance. Differences in health infrastructure, quality of education, and the presence or absence of school feeding and social support programs further contribute to these regional disparities. Understanding these contextual factors is essential for accurately interpreting the findings and for designing tailored, region-specific interventions.

**Table 3:** Summary of subgroup meta-analysis by study region of pooled effect sizes on the academic performance of students in Ethiopia

Study Region	Author, year	Subgroup Pooled Effect Size (95% CI)	Weight (%)	*p* value for Subgroup	I² (%)
Oromia Region	Abebe, Frehiwot (2017) [17]	2.254 (0.657, 3.850)	7.01	0.006	80.90%
	Feye, Dereje (2023) [25]		3.6		
	Haile, Demewoz (2016) [15]		4.77		
	Seyoum, Dejene (2019) [9]		4.66		
	Tamiru, Dessalegn (2016) [28]		5.64		
SNNPR	Abebe, Lulu (2022) [18]	1.542 (−1.107, 4.191)	5.04	0.254	97.80%
	Honja Kabero, Tesfaye (2021) [8]		1.88		
	Senapathy, Marisennayya (2023) [27]		5.52		
	Wolde, Tsedeke (2019) [30]		6.92		
	Zenebe, Mastewal (2018) [31]		6.98		
Amhara Region	Admasie, Amha (2013) [19]	0.631 0.298, 0.963)	7.07	0.044	59.10%
	Asmare, Biachew (2018) [21]		7.09		
	Degarege, Abraham (2022) [16]		5.46		
	Eniyew, Tigist (2019) [24]		1.2		
	Terefe, Birhanu (2021) [29]		2.59		
SWEPR	Asfaw, Agize (2020) [20]	4.490 (4.078, 4.902)	6.98	0	-
Addis Ababa	Bekri Mohammed (2023) [22]	2.148 (1.319, 2.977)	6.32	0	0.00%
	Muluken Ayehu, Solomon (2021) [26]		5.93		
Sidama Region	Desalegn, Tsion A (2021) [23]	2.400 (0.675, 4.125)	5.34	0.000	-
Overall		1.839 (1.040, 2.638)	100	0	

### Subgroup analysis: nutrition vs. WASH interventions

**Fig. 3:**
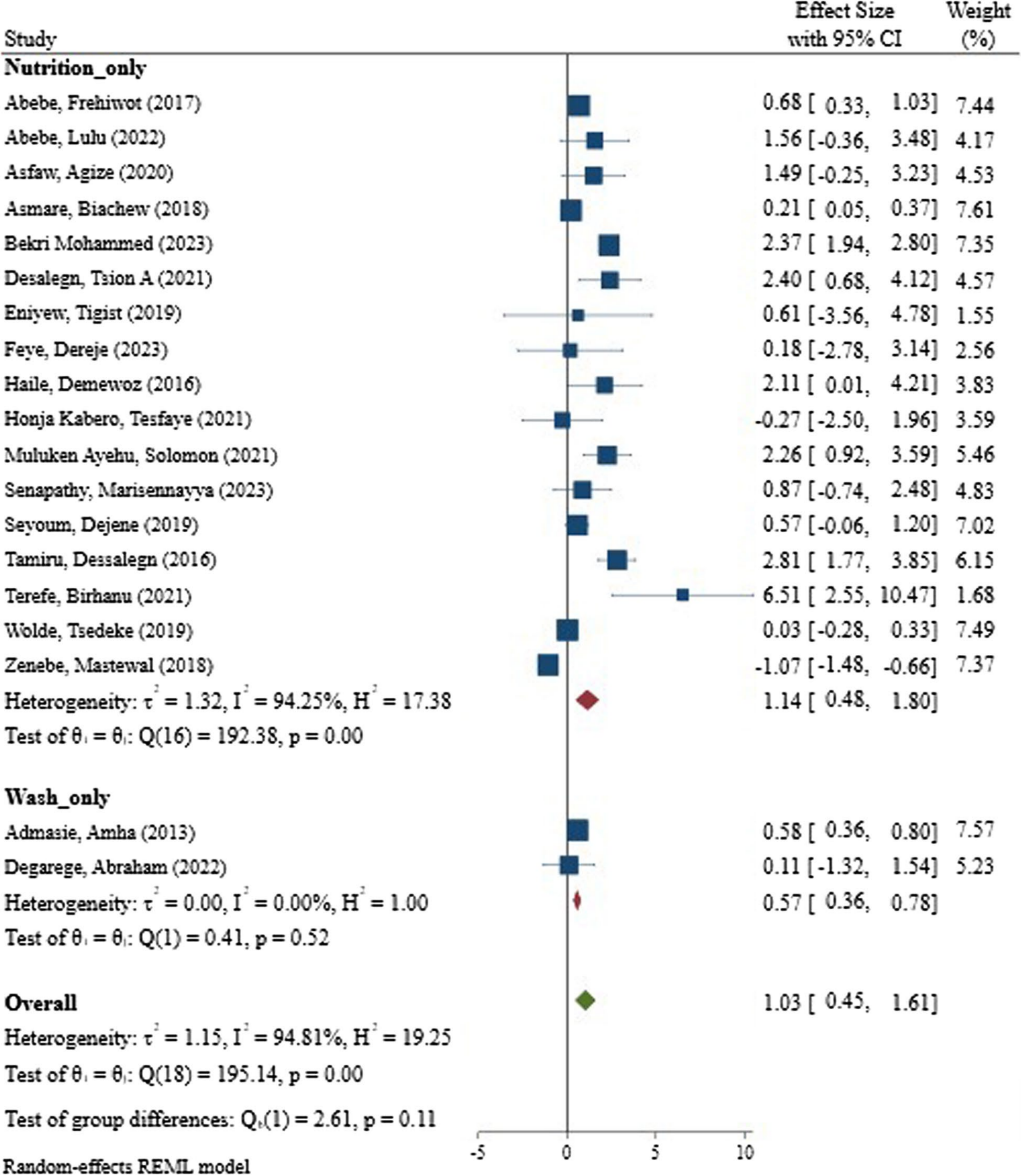
Pooled effect sizes of nutrition-only and wash-only interventions on academic performance of students in Ethiopia: a random-effects meta-analysis

Subgroup analysis was conducted to evaluate the distinct effects of WASH and nutrition interventions on children’s academic performance (Fig. 3). The forest plot presents the results of a meta-analysis comparing the effects of interventions grouped into “Nutrition_only,” “Wash_only,” and an overall combined category. In the “Nutrition_only” group, data from 12 studies—including Abebe, Frehiwot (2017) [17], and Lulitu (2022)—show a wide variation in effect sizes, ranging from 0.68 to 2.47. This section demonstrates substantial heterogeneity, indicated by an I² of 94.25%, τ² of 1.32, and a significant Q statistic (χ² [11] = 192.14, *p* < 0.001). These values suggest that the included studies differ considerably in their findings, possibly due to varying populations, interventions, or methodologies. Despite the variability, most studies in this group report positive effects of nutrition-focused interventions.

In contrast, the “Wash_only” group includes fewer studies—specifically Admasie, Amha (2013) [19] and Degarege, Abraham (2022) [16]—and shows relatively consistent results. The effect sizes here range narrowly between 0.82 and 1.11, and the absence of heterogeneity is confirmed by an I² of 0%, τ² of 0, and a nonsignificant Q statistic (χ² [1] = 0.41, *p* = 0.52). This suggests that the impact of WASH (Water, Sanitation, and Hygiene) interventions is more uniform across studies, possibly reflecting standardized implementation or similar outcome metrics.

The “Overall” analysis aggregates all studies from both groups, yielding a pooled effect size of 1.03 with a confidence interval from 0.45 to 1.61. However, this combined result still reflects high heterogeneity (I² = 94.81%, τ² = 1.15), and the Q statistic remains significant (χ² [13] = 193.54, *p* < 0.001), indicating that diversity among the studies persists even when grouped. Interestingly, the test for group differences (Q = 2.61, *p* = 0.11) does not reveal a statistically significant difference between the two categories, though the nutrition interventions appear to demonstrate a stronger but more variable effect.

### Predictor variables

This systematic review and meta-analysis demonstrated that factors such as sociodemographic characteristics, regional variations, dietary habits, health conditions, and other sociocultural barriers were associated with the academic performance of students in Ethiopia. The subgroup analysis of the predictor or exposure variables revealed greater pooled effect sizes for chronic iodine deficiency (4.490, 95% CI: 4.078, 4.902) [32], the School Feeding Program (1.314, 95% CI: −0.885, 3.514) [23, 24, 31, 33], and food insecurity (2.810, 95% CI: 1.281, 4.339) [22], all of which were statistically significant. These results indicate that these exposures have a stronger association with the outcomes in the studies included in the analysis. Notably, chronic iodine deficiency had the greatest effect size, suggesting that it has a considerable influence on outcomes. However, high heterogeneity was observed across several exposures, particularly in school feeding programs (I² = 94.7%) and intestinal parasitic infections (I² = 90.8%), indicating variability in the study results.

### Nutrition-related factors

The meta-analysis demonstrated that various factors, including underweight, breakfast skipping, chronic iodine deficiency, school feeding programs (SFP), stunting (HAZ), and food insecurity, were associated with the academic performance of students in Ethiopia. Significant associations were observed for certain exposures, such as underweight, skipping breakfast, and food insecurity (*p* < 0.05).

Children who were underweight had a pooled effect size of 0.605 (95% CI: 0.462, 0.749) [20, 26], suggesting a notable link to their academic performance. Two studies indicated that underweight students were less likely to achieve high academic scores. Children’s breakfast skipping has a positive pooled effect size of 3.47 (95% CI: 0.470, 6.470). The pooled effect size of 3.47 indicates that students who skip breakfast are 3.47 times more likely to have poor academic performance than those who regularly eat breakfast. This substantial effect size underscores the significant impact of breakfast skipping on academic outcomes. In this systematic review and meta-analysis, four studies reported that skipping breakfast contributed to academic underperformance.

Chronic iodine deficiency had the strongest association with academic performance, with an effect size of 4.490 (95% CI: 4.078, 4.902) [32]. In contrast, school feeding programs have mixed results, with some studies reporting positive associations and others indicating negative effects, leading to an overall nonsignificant pooled effect size of 1.314 (95% CI: −0.885, 3.514) [23, 24, 31, 33]. Food insecurity, however, exhibited a significant positive association with academic performance, with a large effect size of 2.810 (95% CI: 1.281, 4.339) [22].

**Fig. 4:**
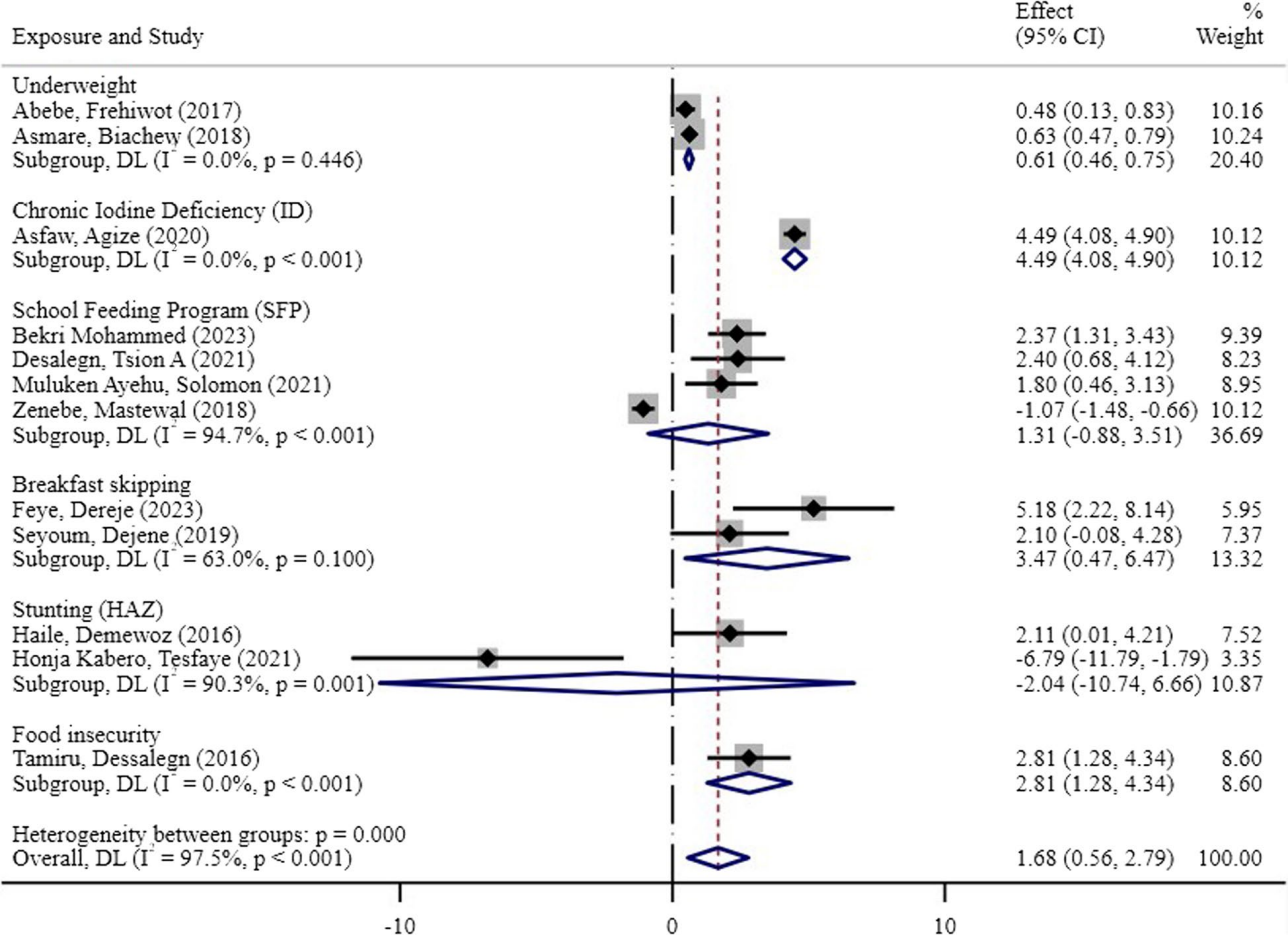
The associations between nutrition-related factors and the academic performance of students in Ethiopia

Owing to significant heterogeneity across studies, as indicated by Cochran’s Q and I² statistics, a random effects model was applied for the analysis. For example, stunting and school feeding programs showed high heterogeneity (I² = 90.3% and I² = 94.7%, respectively), necessitating the use of subgroup analyses to better understand the variability between studies. The overall pooled effect size across all exposures was 1.832 (95% CI: 0.787, 2.876), highlighting the multifactorial nature of academic underperformance among students (Fig. 4).

### WASH-related factors

**Fig. 5:**
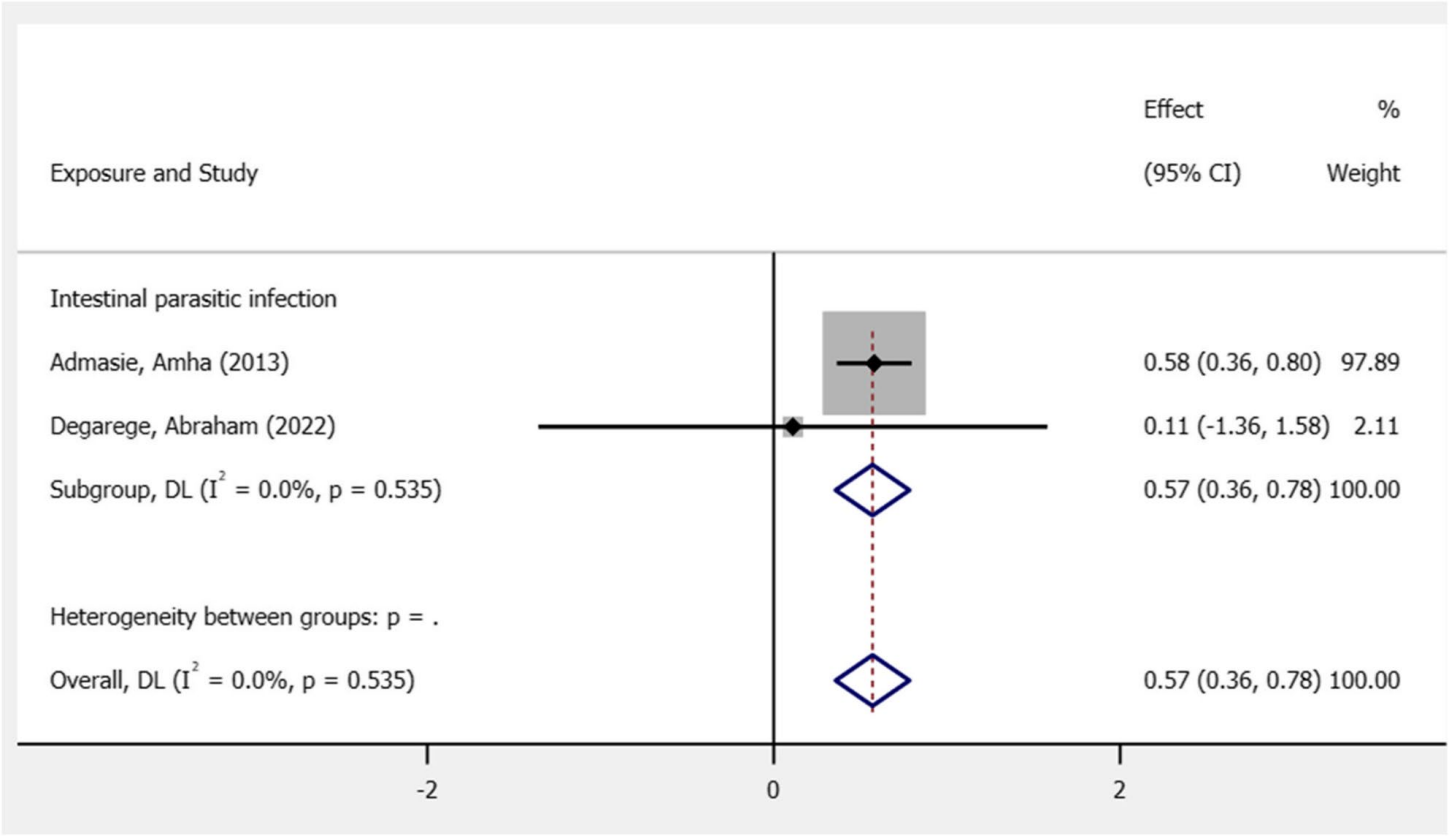
The association between WASH (intestinal parasite infection) and academic performance of students in Ethiopia

A meta-analysis demonstrated that intestinal parasitic infection was associated with academic performance in students in Ethiopia, with a pooled effect size of 0.570 and a 95% CI of 0.357–0.783 [16, 25]. This result indicates that students’ academic performance was negatively affected when they were exposed to parasitic infection, as reported by two studies (Fig. 5).

### Publication bias and sensitivity analysis

The random-effects meta-regression with 19 observations revealed that exposure was significantly associated with academic performance (β = 0.178, 95% CI: [0.040, 0.316], *p* = 0.011. The intercept was also significant (β = 0.733, 95% CI: [0.022, 1.443], *p* = 0.043. The residual heterogeneity was moderate (τ^2^ = 0.2537, I^2^ = 70.25%, and 9.60% of the variance was explained by the model (*R*^2^ = 9.60%). The test for residual homogeneity indicated significant heterogeneity (Q_res_=56.16, *p* < 0.001). Funnel plots and Egger regression tests were used to assess publication bias. The findings revealed an asymmetrical funnel plot and statistically significant publication bias (*p* < 0.05).

The regression-based Egger test [34] for small-study effects, which uses a random-effects model, did not find evidence of small-study effects (β_1_ = 0.40, SE = 0.417, z = 0.96, *p* = 0.337). These findings suggest that publication bias or small-study effects are not statistically significant in this meta-analysis.

The nonparametric trim-and-fill analysis [35] of publication bias, which uses a linear estimator and random effects model, did not impute any additional studies. The pooled effect size remained unchanged at 1.541 (95% CI: 1.180–1.902), indicating that no evidence of substantial publication bias affected the results.

Additionally, the funnel plot suggests potential asymmetry, indicating possible publication bias, where smaller studies with no significant results may be underreported. The majority of points cluster near the top, representing larger, more precise studies, whereas those at the bottom reflect smaller studies with greater variability. The dispersion of points from the central line (RR = 1) highlights heterogeneity among studies, suggesting differences in methodologies, populations, or interventions (Supplemental Fig. 1). This warrants further investigation to confirm and address any biases or inconsistencies.

### Certainty of evidence

The assessments of certainty in the body of evidence for each outcome were conducted via the GRADE approach [13], which focuses on five domains: risk of bias, inconsistency, indirectness, imprecision, and publication bias. The overall certainty of evidence was classified as “High” for both assessed outcomes, supported by robust data from nonrandomized studies and randomized controlled trials (RCTs) (Supplemental Table 1).

## Discussion

This systematic review and meta-analysis revealed that the pooled estimate of the effect size of nutrition and washing on the academic performance of students in Ethiopia was 2.05 (95% CI: 1.26, 2.28) and that these interventions could lead to significant improvements in student outcomes, potentially increasing the proportion of students who perform well academically.

The pooled estimate varied by region and design type. The geographic location where the studies were conducted was the highest in the Oromia region, with 2.25 (95% CI: 0.66, 3.85) in the Oromia region and 2.15 (95% CI: 1.32, 2.98) in Addis Ababa. This might be because the Oromia Region and Addis Ababa might have better access to healthcare facilities, diagnostic tools, or intervention programs, which could increase the reported effect sizes in these regions [36, 37].

The subgroup analysis of studies of academic performance by nutrition-related factors and WASH showed that the pooled effect size varied across different factors. Children who were underweight had a pooled effect size of 0.605 (95% CI: 0.462, 0.749), suggesting a notable link between undernutrition and academic performance. This finding aligns with studies from low-income countries such as India and Bangladesh, which have also demonstrated that undernutrition significantly impairs cognitive function and school performance, underscoring the global relevance of this issue [38, 39].

The pooled effect size of 3.47 revealed that students who skip breakfast are 3.47 times more likely to have poor academic performance than those who regularly eat breakfast. This aligns with findings from a study in Addis Ababa, Ethiopia, which reported that skipping breakfast negatively impacts students’ concentration and learning outcomes [29]. Research in the United States and China has similarly confirmed the adverse effects of skipping breakfast, highlighting its role in poor academic achievement due to reduced energy levels and cognitive function [40–42].

From a scientific point of view, breakfast skipping affects school outcomes by impairing cognitive ability, reducing energy levels, and causing behavioral and emotional challenges. This is because breakfast provides essential nutrients such as iron, calcium, and vitamins, which are crucial for cognitive and physical development [43–46]. Additionally, skipping breakfast leads to lower blood glucose levels, which are vital for optimal brain function [47].

Therefore, ensuring that students have access to a nutritious breakfast should be a priority for parents, schools, and policymakers. School-based breakfast programs, public awareness campaigns, and support for low-income families can play crucial roles in addressing this issue, ultimately improving both the health and academic performance of children [38, 45].

In this systematic review and meta-analysis, chronic iodine deficiency showed the strongest association with academic performance, with an effect size of 4.490 (95% CI: 4.078, 4.902). This result is consistent with findings from studies in Ethiopia, where iodine deficiency disorders are highly prevalent and associated with reduced school performance and cognitive development [48]. A similar trend has been observed in rural China, where iodine deficiency was found to severely affect children’s IQ and learning capacity [49, 50]. Iodine deficiency has long been identified as a major public health issue that can affect cognitive development and academic performance [51]. The scientific basis for this association lies in the role that iodine plays in thyroid hormone synthesis, which is crucial for brain development and function.

School feeding programs have shown mixed results, with an overall pooled effect size of 1.314 (95% CI: −0.885, 3.514), which is statistically insignificant. This may be attributed to varying factors. While some Ethiopian studies, such as Bekri Mohammed (2023) [22], Desalegn, Tsion A (2021) [23], and Muluken Ayehu, Solomon (2021) [26], report positive impacts on school attendance and concentration, others highlight challenges, including poor food quality and insufficient rations, which undermine the effectiveness of these programs Zenebe, Mastewal (2021) [31]. Globally, school feeding programs in sub-Saharan Africa and South Asia exhibit similar variability, with outcomes largely dependent on the quality and consistency of program implementation [52].

In this systematic review and meta-analysis, food insecurity was significantly positively associated with academic performance, with an effect size of 2.810 (95% CI: 1.281, 4.339). This finding is supported by studies in Kenya and Ghana, which have demonstrated that food-insecure households experience challenges in maintaining children’s school performance due to limited access to nutritious meals [53, 54].

Although the funnel plot appeared asymmetrical, suggesting potential publication bias, statistical tests including Egger’s regression (*p* = 0.337) and the trim-and-fill method did not confirm the presence of small-study effects or missing studies. This discrepancy may be due to the limited power of statistical tests in meta-analyses with fewer than 20 studies. In such cases, visual inspection of funnel plots can be subjective, and asymmetry may result from heterogeneity in study populations, methodologies, or interventions rather than true publication bias. Therefore, while potential bias cannot be fully ruled out, the statistical evidence does not indicate a significant threat to the validity of the pooled results.

### Limitations of this review and meta-analysis

The evidence in this review and meta-analysis has several key limitations. Regional variations, such as higher effect sizes observed in Oromia than in SNNP, may reflect differences in local conditions and intervention implementation rather than the true effect. Additionally, heterogeneity across study designs, populations, and outcome measures limits the comparability and reliability of the findings. The majority of the included studies were cross-sectional in design, which restricts our ability to infer causality between exposure and outcome variables. As such, the associations identified should be interpreted with caution. Mixed outcomes for interventions, such as school feeding programs, underscore variability in program quality. The lack of data on long-term impacts and the potential for publication bias further limit the generalizability of the results. High-quality, longitudinal studies are needed to address these gaps.

Moreover, although publication bias was assessed using funnel plots and statistical tests (e.g., Egger’s test), the potential for bias remains. Studies with null or negative findings may have been underreported or unpublished, potentially affecting the overall results. While study quality was evaluated using standardized appraisal tools, subjective judgment during the critical appraisal process may have introduced additional bias. Additionally, the reliance on published studies may have excluded research with insignificant or negative findings, further contributing to potential publication bias. Furthermore, language restrictions and the exclusion of gray literature may have led to the omission of relevant data. Inconsistencies in data extraction and coding, despite efforts to standardize the process, could have impacted the overall synthesis of the findings.

## Conclusion

This systematic review and meta-analysis demonstrated that nutrition and WASH interventions play an important role in improving the academic performance of school-aged children in Ethiopia. The pooled effect size indicates that children who benefit from adequate nutrition, regular breakfast intake, sufficient iodine consumption, and reduced exposure to intestinal infections tend to achieve better educational outcomes. However, considerable variability exists across regions and intervention types, highlighting the need for context-specific strategies. Strengthening integrated school-based nutrition and WASH programs, prioritizing underserved areas, and ensuring consistency in program delivery may yield substantial improvements in both health and learning. Future research should prioritize longitudinal and experimental designs to establish causal pathways and assess long-term, cost-effective solutions that support child well-being beyond academic achievement.

### Implications for practice, policy, and future research

Future research should aim to address current evidence gaps by examining the long-term effects of nutrition interventions on both academic performance and health outcomes. In addition to quantitative evaluations, qualitative studies are essential to explore students’ lived experiences, perceptions, and the cultural relevance of school-based nutrition programs. For instance, understanding how students and families perceive the quality and acceptability of school meals can provide valuable context for improving program design and implementation.

Furthermore, future research should assess the cost-effectiveness of these interventions across diverse regions and investigate the contextual factors that influence their success or failure. Mixed-methods and longitudinal study designs would be particularly effective in capturing both measurable impacts and deeper contextual insights to inform policy and practice.

From a practical standpoint, schools should prioritize the implementation of effective feeding programs and address persistent issues such as iodine deficiency through sustained efforts. This includes educating students and families about the importance of iodized salt, encouraging regular breakfast consumption, and fostering collaboration with parents and local health workers. Policymakers should focus on expanding access to high-quality interventions in underserved regions and ensuring their consistent delivery. Additionally, future research should explore causal relationships through longitudinal designs and identify region-specific barriers to implementation.

Expanding the evidence base in these areas will support the development of tailored, context-sensitive strategies to improve student health and educational outcomes. Additionally, although this study focused on academic performance as the primary outcome, future research should evaluate broader dimensions of child well-being that may be influenced by nutrition and WASH interventions. These may include physical health indicators (e.g., growth metrics, infection rates) and psychological outcomes such as emotional resilience, motivation, and cognitive development. Adopting a multidimensional framework would offer a more holistic understanding of intervention impacts and strengthen the evidence base for integrated policy and programmatic strategies.

## Supplementary Information

Supplementary Material 1: Table S1. Summary of certainty assessment via the GRADE Pro for Nutrition and WASH interventions on the academic performance of students in Ethiopia (*n* = 19).

Supplementary Material 2: Table S2. PRISMA 2020 for Abstracts Checklist.
Table S3. PRISMA 2020 Checklist for Reporting the Systematic Review and Meta-Analysis.

Supplementary Material 3: Fig S1. Funnel plot illustrating the relationship between the standard error of the log risk ratio (SE[log(RR)]) and the risk ratio (RR).

## Data Availability

All relevant data are included in the paper and its supporting information files.
