# Supplementary material for: Associations between exposure to nutrition, WASH interventions and children’s academic performance in Ethiopia: a systematic review and meta-analysis
Source: BMC Public Health. 2026 Jan 12;26:798. doi: 10.1186/s12889-025-26107-4 (PMC12961871; doi:10.1186/s12889-025-26107-4)
Supplement: Supplementary file 1 — Supplementary Material 1: Table S1. Summary of certainty assessment via the GRADE Pro for Nutrition and WASH interventions on the academic performance of students in Ethiopia (n = 19). [file 12889_2025_26107_MOESM1_ESM.docx]

**Supplemental Table 1.** Summary of Certainty Assessment using GRADE pro for Nutrition and WASH Interventions on the Academic Performance of Students in Ethiopia (n = 19)

| Certainty assessment | | | | | | | Summary of findings | | | | |
| --- | --- | --- | --- | --- | --- | --- | --- | --- | --- | --- | --- |
| Participants (studies) Follow-up | Risk of bias | Inconsistency | Indirectness | Imprecision | Publication bias | Overall certainty of evidence | Study event rates (%) | | Relative effect (95% CI) | Anticipated absolute effects | |
|  |  |  |  |  |  |  | With Academic performance | With Nutrition or WASH Predictor variables |  | Risk with Academic performance | Risk difference with Nutrition or WASH Predictor variables |
| New outcome (follow-up: range 1 months to 12 months; assessed with: Adjusted odd ratio) | | | | | | | | | | | |
| 15474 (15 non-randomised studies)^a^ | not serious | not serious | not serious | not serious | none | ⨁⨁⨁⨁ High | 4198/7836 | 3638/7638^a^ | Rate ratio 2.17 (1.01 to 3.33) | Study population | |
|  |  |  |  |  |  |  |  |  |  | 4198/7836 | |
|  |  |  |  |  |  |  |  |  |  | Low bias | |
|  |  |  |  |  |  |  |  |  |  |  |  |
| New outcome (follow-up: range 1 months to 12 months; assessed with: score) | | | | | | | | | | | |
| 3552 (4 RCTs) | not serious | not serious | not serious | not serious | none | ⨁⨁⨁⨁ High | 852/1776 (48.0%) | 924/1776 (52.0%) | OR 2.17 (1.01 to 3.33) | Study population | |
|  |  |  |  |  |  |  |  |  |  | 852/1776 (48.0%) | 187 more per 1,000 (from 2 more to 275 more) |
|  |  |  |  |  |  |  |  |  |  | Low bias | |
|  |  |  |  |  |  |  |  |  |  |  |  |

**CI:** confidence interval; **OR:** odds ratio

#### Explanations

1. Nutrition and WASH
