# Supplementary figures and images for: Associations between exposure to nutrition, WASH interventions and children’s academic performance in Ethiopia: a systematic review and meta-analysis
Source: BMC Public Health. 2026 Jan 12;26:798. doi: 10.1186/s12889-025-26107-4 (PMC12961871; doi:10.1186/s12889-025-26107-4)

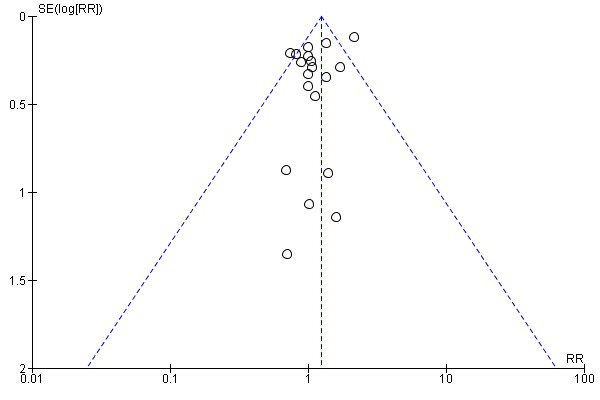

Supplement: Supplementary file 3 — Supplementary Material 3: Fig S1. Funnel plot illustrating the relationship between the standard error of the log risk ratio (SE[log(RR)]) and the risk ratio (RR). [file 12889_2025_26107_MOESM3_ESM.tiff]
